# Effects of Dietary *Melissa officinalis* and Feeding Strategy on Growth and Physiological Responses in Hybrid Red Tilapia

**DOI:** 10.1155/anu/2291078

**Published:** 2025-10-11

**Authors:** Mohamed F. Abdel-Aziz, Eman M. S. Shaheen, Shymaa M. Shalaby, Ashraf Y. El-Dakar, Mohamed Abdul Gouad, Aseel F. Ashour, Mageed M. Abdrabou, Mahmoud Mabrok, Afaf N. Abdel Rahman

**Affiliations:** ^1^Department of Aquaculture and Biotechnology, Faculty of Aquaculture and Marine Fisheries, Arish University, Arish, Egypt; ^2^Zoology Department, Faculty of Science, Benha University, Benha, Egypt; ^3^Department of Aquaculture, Faculty of Fish Resources, Suez University, Suez, Egypt; ^4^Department of Aquatic Animal Medicine, Faculty of Veterinary Medicine, Suez Canal University, Ismailia 41522, Egypt; ^5^Department of Microbiology and Parasitology, Faculty of Veterinary Medicine, King Salman International University, Ras Sudr 46612, Egypt; ^6^Department of Aquatic Animal Medicine, Faculty of Veterinary Medicine, Zagazig University, P.O. Box 44511, Zagazig, Egypt

**Keywords:** aquaculture industry, feeding strategies, fish physiology, health status, lemon balm, red tilapia

## Abstract

Generally, the inadequate feeding of aquatic organisms can mitigate stress and disease vulnerability. In contrast, overfeeding has worsened the water quality in addition to the high feeding cost. Hence, the present study was performed to evaluate two feeding regimes (alternate-day feeding [ADF] and daily feeding [DF]), plus dietary supplementation with *Melissa officinalis* (lemon balm leaves [LBL]) on growth, body composition, digestion, and hemato-physiological status of hybrid red tilapia (*Oreochromis* spp.). Fish (*n* = 180) with an average weight of 18.14 ± 0.48 g were distributed into six groups (30 fish/group; 3 replicates/group; 10 fish/replicate) in a 2 × 3 factorial feeding trial for 60 days. The groups were ADF + LBL0, ADF + LBL1, ADF + LBL2, DF + LBL0, DF + LBL1, and DF + LBL2. The LBL was added to the basal diet at the doses of 0%, 1%, and 2% (LBL0, LBL1, and LBL2), respectively. The outcomes revealed that ADF achieved the best feed conversion ratio (FCR) (1.68), but the growth did not show substantial variation between ADF and DF. The LBL1 supplementation enhanced final body weight (FBW) (33.16 g), total weight gain (TWG) (14.20 g/fish), and specific growth rate (SGR) (0.93 %/day). The interaction between LBL and feeding strategies showed that the ADF + LBL1 had the best FCR (1.31). Crude lipid and ash content substantially changed (*p* < 0.05) by feeding regimes and LBL addition. The DF + LBL0 group had the highest lipid (32.08%) and the lowest ash content (18.28%). Dietary LBL2 significantly increased (*p* < 0.05) hemoglobin (Hb) (8.30 g/dL), hematocrit (Hct) (26.25%), and white blood cell (WBC) count (419.44 × 10^3^/mm^3^). Moreover, the ADF regime significantly decreased (*p* < 0.05) glucose level (47.83 ng/L); meanwhile, the DF regime notably increased amylase activity (47.66 U/g protein). Our conclusion suggested that feeding hybrid red tilapia with an ADF schedule with a diet enriched with LBL can improve growth, feed utilization, and physiological response. This offers an avenue to assist the aquaculture sector and lower feed expenses.

## 1. Introduction

Considering feed costs range from 60% to 70% of overall operating expenses, feed is a critical component of modern aquaculture's sustainability and profitability [1]. It also has a significant impact on the immune system and disease resistance of the fish [2, 3]. In addition, the availability, management, and composition of feed affect the growth of fish in aquaculture [4]. Thus, feed management and quality are both vital and quite sensitive [5].

Certain fish species may go through periods of natural fasting in their native habitats due to a shortage of nutrients, seasonal migration, or reproduction [6]. In this regard, the fish that receive suboptimal feeding or food deprivation suffer from high stress, reduced growth, and vulnerability to diseases. This is because they consume the body's stored nutrients to sustain their life, which results in physical and behavioral alterations [4, 7]. Conversely, overfeeding has caused the water quality to drastically worsen, which is detrimental to fish growth [8]. Consequently, feeding routines should be adjusted to enhance feed efficiency and fish growth by maximizing protein usage and digestion [9]. Also, the proper feeding can lower aquaculture production costs and prevent water quality deterioration resulting from overfeeding [10, 11].

Fish species are among the factors that affect feeding frequency and rate [12]. Tilapia and its hybrids have been a major factor in the expansion of aquaculture worldwide. The yearly production of tilapia culture is predicted to reach 7.3 million tons by 2030 [13]. One of the most significant species for commercial aquaculture is the hybrid red tilapia (*Oreochromis* spp.). Its appeal is mostly due to its saline tolerance, ease of adaptation to confinement and cultivation conditions, and attractive color [14]. Few reports on the feeding frequency and nutritional requirements of the hybrid red tilapia exist. Because tilapia that are fed every 2–3 h consume more food than their gut capacity, some food exits the stomach undigested. Moreover, feeding tilapia day after day can be done without negative effects on growth [15].

On the other hand, medicinal plants or herbs are one of the key factors for promoting growth rate, nutritional efficiency, and physiological state in aquaculture [16–18]. Lemon balm (*Melissa officinalis*), a member of the *Lamiaceae* family, is considered one of the important medicinal plants which had several properties [19]. It contains several compounds, including flavonoids (such as quercitrin, ramnocitrin, luteolin, and glucuronopyranoside), terpenoids (neural geranyl acetate and tannins), and essential oils, which act as an antioxidant and antimicrobial agent [20, 21].

Lemon balm can increase the immune response and growth of rainbow trout (*Oncorhynchus mykiss*) [22]. Moreover, lemon balm can alleviate stress during the transportation of sea bass (*Lateolabrax japonicus*) [23]. As far as we comprehend, no work has been carried out to investigate the dual effect of dietary lemon balm leaves (LBL) and short-term starvation on fish health. It is hypothesized that alternate-day feeding (ADF) combined with dietary LBL would improve growth and physiological parameters while enhancing feed efficiency in hybrid red tilapia. Thus, the current study is the first of its kind to assess the LBL as a supplementary feed under the deprivation and refeeding system on the growth rate, body composition, biochemical and hematological variables, and gut digestive enzymes of red hybrid tilapia.

## 2. Materials and Methods

### 2.1. Statement of Ethics and Location of Experiment

All animal welfare considerations taken, including efforts to minimize suffering and distress, use of analgesics or anesthetics, or special housing conditions, were in strict accordance with the recommendations in the Guide for the National Institute of Oceanography and Fisheries (NIOF) Committee for Ethics Care and Used Aquatic Animal (NIOF-IACUC) and approved with the code number (NIOF-AQ1-F-22-R-026). This study was conducted in the Fish Nutrition Lab, Faculty of Aquaculture and Marine Fisheries, Arish University. Experimental fish were obtained from fish farming at Arish University, Arish, Egypt.

### 2.2. Tested Diets Preparation

LBL in dried form was obtained from a local herbal product market in Fayoum, Egypt. This powder was added to the ingredients of the basal diet at 0%, 1%, and 2% to create three iso-nitrogenous and iso-caloric tested diets (LBL0, LBL1, and LBL2), respectively. The total flavonoids and phenolic compounds of the LBL were 2.40 μg/g extract and 12.57 mg/g extract, respectively, according to a previous study [24]. Diets were established following the guidelines of NRC [25] to suit the nutritional requirements of fish. The components were mechanically milled and well blended with some water until a dough was obtained. After that dough was pelletized by a mincer with a die of 2 mm. Pellets were sun-dried and stored at 4°C until used. As per the AOAC [26] protocol, the chemical analysis of the basal diet was carried out (Table 1).

### 2.3. Fish Rearing and Experimental Design

Red hybrid tilapia juveniles were transferred to the work site in a plastic tank supplied with aeration. Juveniles were adapted to rearing unit conditions for 2 weeks. After the adaptation period, fish were randomly divided into 18 plastic tanks with a water volume of 45 L, with a density of 10 fish/tank (3 tanks/group). Tanks were continuously aerated with six air pumps (220 V, 50 Hz, 5 W) to produce around 4 L of oxygen/min. Fish were cultured in brackish water with a 3-ppt salinity and fed two times daily at 10 a.m. and 5 p.m. with a feeding rate of 4% of biomass. The initial body weight (IBW) of fish was 18.14 ± 0.48 g.

A 2 × 3 factorial feeding trial for 60 days was carried out with two strategies of feeding (ADF) and (daily feeding [DF]). Fish were divided into six groups (30 fish/group) in triplicates; each group included three tanks (10 fish/tank). The treatments were involved ADF + LBL0, ADF + LBL1, ADF + LBL2, DF + LBL0, DF + LBL1, and DF + LBL2 groups. Fish were fed the tested diets under two feeding strategies. Feeding rates were adjusted every 15 days according to changes in their body weight, and the remains of uneaten feed and fish waste were removed from the tank bottom using a small suction pump. The water exchange rate was 50% of the water volume weekly.

### 2.4. Examination of Water Quality

Throughout the 60 day-trial, the water variables were routinely checked. Water temperature and pH were determined daily by the pH-meter (HI2211) of HANNA instruments, USA. Dissolved oxygen (DO, mg/L) was recorded weekly by oxy-meter (HI98198; Hanna Instruments, USA). Total ammonia (TAN, mg/L) was measured every week by a multi-parameter ion analyzer (Hanna Instruments, USA).

### 2.5. Determination of Growth, Feed Utilization, and Survival Assays

Fish were weighed at the termination of the trial as final body weight (FBW), and the following metrics were determined as follows:

  $$\begin{array}{c} \text{Total weight gain }\left(\text{TWG},g/\text{fish}\right)=\frac{\text{FBW }\left(g\right)/\text{tank}-\text{IBW }\left(g\right)/\text{the same tank}}{N}, \end{array}$$

  $$\begin{array}{c} \text{Average daily gain }\left(\text{ADG},g/\text{fish}/\text{day}\right)=\frac{\text{TWG }\left(g/\text{fish}\right)}{\text{Experimental days}}, \end{array}$$

  $$\begin{array}{c} \text{Specific growth rate }\left(\text{SGR},\%/\text{day}\right)=\;100\times \frac{\text{ln FBW }-\text{ln IBW}}{\text{Experimental days}},\text{where Ln is the natural logarithm}, \end{array}$$

  $$\begin{array}{c} \text{Feed conversion ratio }\left(\text{FCR}\right)=\;\frac{\text{Total feed intake }\left(g\right)}{\text{TWG }\left(g/\text{fish}\right)}, \end{array}$$

  $$\begin{array}{c} \text{Survival rate }\left(\text{SR},\%\right)=\;100\times \frac{\text{Final fish number }}{\text{Initial fish number}}. \end{array}$$

### 2.6. Sampling

At the end of the feeding trial, all fish from each tank were anesthetized with MS-222 tricaine methane sulfonate, 0.1 g/L (Sigma, USA) as reported by Yue et al. [27]. Two fish from each tank (6/group) were randomly taken and frozen at −20°C for body composition analysis. Three fish from each tank (9/group) were randomly sampled for blood and intestinal tissues. Blood was taken from the caudal blood vessels using 1 mL syringes. The obtained blood was divided into two tubes, one of which contained 1 mL anticoagulant (EDTA) for hematological assessment. Other tubes were without EDTA, followed by centrifugation at 1075 ×*g* for 10 min at 4°C to obtain serum for biochemical assays. After collecting the fish's blood, the fish were euthanized, and the entire intestine was taken for digestive enzyme evaluation.

### 2.7. Body Composition Assays

The chemical composition analyses of the whole-body fish (6/group) followed the described methods [28]. Moisture was determined by oven drying at 105°C until constant weight for 16–24 h. Crude lipid was analyzed by ether extraction using a Soxtec System with petroleum ether at 60–80°C boiling, while crude protein (nitrogen X 6.25) was analyzed by the Kjeldahl method. Moreover, ash was estimated by the combustion of a 1 g sample at 550°C for 4 h in a muffle furnace.

### 2.8. Hematological and Biochemical Assays

The hematological profile involving red blood cells (RBCs) count, hemoglobin (Hb), hematocrit (Hct), and white blood cells (WBCs) count was estimated using the fully automatic hematological analyzer (Genrui-KT-6400, China). Serum alanine aminotransferase (ALT; REF:263002) and aspartate aminotransferase (AST; REF:292001) activity, and the level of glucose (REF:250002) and cholesterol (REF:230002) were analyzed spectrophotometrically using spectrum diagnostics kits of Egyptian Company for Biotechnology, Obour City, Egypt, employing prior procedures [29–31]. Besides, blood alpha-antitrypsin was determined according to the described methods by Ruiz-Duque et al. [32].

### 2.9. Digestive Enzymes Investigation

The whole intestinal samples were removed, weighed, and homogenized in plastic pistils in phosphate buffer saline at a ratio of 1:10 [33]. Each sample was centrifuged (4°C/13,000 × *g*) for 3 min. Following that, the supernatant was moved to ice-filled microtubes to assess the activity of digestive enzymes (lipase and amylase). Using the Kruger [34] approach, each sample's total protein content was estimated to calculate the enzyme activity (U/g protein). Bradford reagent (980 μL) dissolved in distilled water (proportion 1:5) was added to microtubes, and then the homogenate samples that were diluted in phosphate buffer saline (proportion 1:500) were admixed. Following a 10-min incubation period, the absorbance of each solution at 595 nm was measured using a microplate reader (Biotek Business, USA). The techniques described by Worthington [35] and Bernfeld [36] were implemented for analysis of the intestinal lipase and amylase activity, respectively.

### 2.10. Data Analysis

The Kolmogorov–Smirnov and Bartlett tests were implemented to check the data's homogeneity and normality, and the results verified these assumptions. Consequently, one-way and two-way analysis of variance (ANOVA) were applied to analyze the obtained data using the SPSS statistical package program v.17 (SPSS, 2008). Tukey's HSD test was employed to evaluate the variations among treatments at a significance level of 0.05 (*p* < 0.05). In addition, the sample size was calculated through the following website: http://www.biomath.info/power/prt.htm.

## 3. Results

### 3.1. Water Quality Variables

Table 2 demonstrates that there were no significant alterations in the values of the water temperature, pH, DO, and TAN among groups.

### 3.2. Growth, Feed Utilization, and Survival

Table 3 shows nonsignificant differences in FBW, total weight gain (TWG), average daily gain (ADG), and specific growth rate (SGR) between fish fed under ADF and DF strategies. However, feed conversion ratio (FCR) was significantly better (*p* < 0.05) in fish-fed under ADF than DF. Concerning the effect of dietary LBL alone, fish-fed dietary LBL1 exhibited substantial augmentation (*p* < 0.05) in the FBW, TWG, ADG, and SGR values compared with that of LB0; however, dietary LBL2 did not change these metrics. Despite the lack of significant differences in FCR value, the best FCR was obtained by dietary LBL1. Fish displayed 100% survival rate (SR) throughout the trial. Interaction between feeding strategies and LBL-supplemented diets presented substantial differences (*p* < 0.05) among groups in all variables. The ADF + LBL1 and DF + LBL1 groups showed significantly the highest values over the other groups in the FBW, TWG, ADG, and SGR values. The lowest FCR was recorded with ADF + LBL1 (1.31), while the DF + LBL2 group had the worst result (2.72).

### 3.3. Body Composition

Table 4 exhibits a nonsignificant alteration in the values of the moisture, crude lipid, crude protein, and ash of the whole body by the feeding strategy. Conversely, dietary LBL supplements exerted a significant influence (*p* < 0.05) on the body lipid and ash, where the LBL2 group revealed the lowest crude lipid content and the highest ash content. In addition, the maximum crude protein content was noted in dietary LBL1 and LBL2 groups compared to the LBL0 group, but the increase was insignificant. Additionally, the feeding system's interaction with LBL-supplemented diets revealed negligible variations in moisture and protein content between the groups. While crude lipid and ash showed substantial changes (*p* < 0.05). The DF + LBL0 group had the highest lipid and the lowest ash content.

### 3.4. Hematological Variables

The feeding schedule did not significantly affect hematological markers, as shown in Table 5. On the contrary, a significant increase (*p* < 0.05) in Hb, Hct, and WBCs was found with increasing doses of LBL, and the dietary LBL2 reported the highest values. In the same trend, the interaction between feeding strategies and dietary LBL showed significant differences (*p* < 0.05) in Hb, Hct, and WBCs values. The ADF + LBL2 and DF + LBL2 groups exhibited the best findings.

### 3.5. Biochemical Variables

Table 6 reveals that the activity of ALT, AST, and cholesterol, as well as the values of alpha-antitrypsin, are not affected by feeding strategies or LBL supplements. In contrast, glucose levels were significantly altered by the feeding strategy, where ADF markedly decreased glucose (*p* < 0.05). The feeding system's interaction with LBL-supplemented diets showed that ADF groups had significantly lower glucose (*p* < 0.05) than those of DF groups.

### 3.6. Digestive Enzyme Activity

Table 7 reveals significantly more elevation (*p* < 0.05) in the activity of amylase enzyme in the DF than in ADF; however, the lipase activity did not change. The dietary LBL had no significant impact on these enzyme levels, but the activity of digestive enzymes gradually increased with increasing LBL levels. Concerning the interaction, the activity of digestive enzymes increased significantly (*p* < 0.001) in the DF groups over the ADF groups, with the DF + LBL2 group recording the highest value.

## 4. Discussion

For fish growth and welfare in the aquaculture industry, feeding frequency must be carefully adjusted [37]. In addition, medicinal plants as feed supplements are essential for enhancing the growth, nutritional effectiveness, and physiological status of aquatic organisms. To address this concern, the response of hybrid red tilapia juveniles to two levels of LBL (1% and 2%) under two distinct feeding schemes (ADF and DF) regarding growth, digestive capability, and physiological condition was evaluated.

### 4.1. Growth and Feed Utilization

Fish quality, growth, and productivity are significantly impacted by the physicochemical characteristics of culturing water. Deterioration of water quality may thereby affect aquatic life [38]. In this study, water quality indices' means were not significantly varied among treatments. The outcomes indicated that neither the feeding regimes nor dietary LBL influenced the water variables. The latter was found to be within the acceptable limits for tilapia culture according to EPA [39] and El-Sayed [40].

Notably, feeding schedules are one of the tools that reduce feed costs due to feeding restrictions and improve feed conversion due to the reduction of uneaten feed [41]. Our findings verified a marked improvement in the FCR in the ADF regime. Prior reports [15, 42] supported our findings. In the same trend, Bjørnevik et al. [43] recorded that feeding costs can be drastically reduced without compromising the biomass growth of Atlantic cod (*Gadus morhua*) by applying feeding on alternate days. The positive effects of the ADF system may be attributed to the phenomenon of compensatory growth, which enhances appetite and feed utilization, hence achieving better FCR [44]. Moreover, short-term starvation causes muscle and visceral fats to mobilize as energy sources, and when feed is supplied, muscle lipid is replaced with water. This results in a rapid increase in muscle glycogen and lipid [15, 45]. In addition, nutrient efficiency in the intestine increases during the fasting period, leading to a more efficient uptake of nutrients and their absorption when feeding after this time [46].

Interestingly, LBL-supplemented diets affected fish performance significantly regardless of the feeding system, where dietary LBL1 enhanced growth metrics. Our results followed a previous report [22, 47] on rainbow trout and Nile tilapia-fed dietary lemon balm. This positive impact may be due to the richness of LBL with phytochemicals as flavonoids and phenolic compounds [48], together with its aromatic flavor, which increases diet palatability. Also, these compounds have antioxidant, antimicrobial, and anti-inflammatory potential, which is reflected in growth [20].

Despite the improved FCR in the LBL1 treatment, increasing the level of LBL (2%) led to an elevation of FCR, especially in the DF + LBL2 group. This finding may be due to the presence of antinutritional components in LBL that have an adverse influence on FCR. In particular, high concentrations of these compounds in LBL may decrease palatability, feed intake, digestion, or nutrient absorption, which would lower feed utilization. Hence, LBL at a low level (1%) may boost digestion, resulting in augmented growth. While the higher concentrations (2%) may make the antinutritional elements more noticeable, which decreases feed utilization, leading to digestive issues and lowered growth. These mechanisms still need further investigation.

### 4.2. Physiological and Hematological Responses

Blood indicators are the reflection of fish health and immunological status [49]. Findings of the hematological profile showed fish fed the LBL2 diet had high values of Hb, Hct, and WBCs under the two schedules. These outcomes indicated a better health and immune status of fish exerted by LBL. These results were in line with previous reports on rainbow trout and Nile tilapia [22, 47, 50].

It is possible to use blood glucose levels as a signal for the physiological state of fish. Its level is affected by different factors such as environmental conditions, stress, and feeding frequency [51, 52]. In this study, the DF schedule notably increased blood glucose levels higher than the ADF system. This result was alien with the increase in the amylase activity under the same system (DF). Amylase is an indispensable indigenous digestive enzyme, which primarily breaks down starch into a simple sugar (glucose) used as energy [53]. Consequently, more starch is metabolized when amylase activity elevates, which may raise blood glucose levels [54]. On the other hand, the blood glucose value is decreased under the ADF routine. This outcome was because of its use as an extra energy source for metabolic functions during short fasting and a reduction in the available glycogen reserves [55]. To maintain glucose levels for the vital organs such as the brain during fasting, the body initiates a sequence of hormonal changes, mainly by raising corticosteroids (such as cortisol) and glucagon, and decreasing insulin of fish [55–57]. The latter promotes glucose uptake, while corticosteroids promote glucose release and decrease its uptake in peripheral tissues [58]. These hormones function antagonistically to maintain blood glucose homeostasis, but further study is needed on this avenue. Similar findings [59, 60] in red tilapia and glass eel (*Anguilla bicolor bicolor*) confirmed our results.

### 4.3. Digestive Function

Digestive enzymes play a significant role in the hydrolysis of fat, carbohydrate, and protein, therefore boosting the absorption of nutrients [61, 62]. In our investigation, lipase and amylase activity increased under the DF schedule in fish-fed LBL diets compared with ADF. Presumably, continuous feeding is a main factor in stimulating the secretion of digestive enzymes [63]. According to Ma et al. [64] and Liang et al. [65], the DF stimulates cholecystokinin (*CCK*) that promotes the release of the digestive enzyme in fish. *CCK* is an essential peptide from enterocytes that affects digestion and appetite signal activation, and its expression is influenced by DF patterns [66]. In contrast, the ADF (short-term fasting) decreased secretion of digestive enzymes in this study, which may be due to the downregulation of CCK expression as reported by Le et al. [67]. The length and frequency of starvation, as well as the particular fish species and their dietary patterns, can all affect how much these changes occur. However, this pathway requires further research.

### 4.4. Nutritional Implications

Concerning body composition, the body content of lipid notably decreased, and ash elevated with LBL level. The potential of lemon balm to reduce serum lipoprotein and triglycerides, and affect fatty acid biosynthesis may be the explanation. This is may be attributed to LBL contains volatile terpenoids, which can inhibit lipid biosynthesis and the formation of cholesterol in bile, alongside its antioxidant properties [68, 69]. In addition, the terpenoids of lemon balm can inhibit lipogenesis via suppressing sterol regulatory element binding protein 1c (*SREBP-1c*), activation of AMP-activated protein kinase (*AMPK*), and lowering lipogenic enzyme activity [70, 71]. *SREBP-1c* is a transcription-factor protein that mostly involved in controlling lipid metabolism, specifically the production of cholesterol and fatty acids [72]. While *AMPK* stimulates the catabolic (energy-producing) mechanisms [73].

### 4.5. Limitations of the Study

This work solely relies on the growth, body composition, hemato-biochemical, and digestive responses of hybrid red tilapia under feeding practices and dietary incorporation of medicinal plant (LBL). However, numerous limitations must have to be addressed. First, there is a lack of feed intake/palatability data, which can explain feed conversion variation. Another limitation is the absence of evaluating other health biomarkers like stress indicators (e.g., cortisol, malondialdehyde), gene expression (molecular-level), and chemical or histological analysis to support observed physiological effects. These aspects draw attention to areas that require more research to reinforce the study's conclusions.

## 5. Conclusion

This study demonstrates that 1% lemon balm leaf (LBL1) inclusion combined with ADF enhances feed efficiency and physiological resilience in hybrid red tilapia. This strategy offers a promising approach to reducing feed costs while supporting fish health, though further studies incorporating molecular and histological analysis are recommended.

## Figures and Tables

**Table 1:** Ingredients of basal diet and a proximate chemical composition (basis dry matter; %).

Ingredients	%
Fish meal 65% CP	17.00
Soybean meal 44% CP	32.00
Yellow corn 8% CP	36.00
Wheat bran 14% CP	10.00
Starch	1.70
Soy oil	3.00
Vitamins and mineral mixture^a^	0.30
Total	100

**Chemical composition**	**%**

Dry matter	90.42
Crude protein	29.40
Ether extract	6.35
Crude fiber	4.12
Ash	6.65
Nitrogen-free extract^b^	53.38
Gross energy (kcal/kg)	4627.12

⁣^a^Vitamins and mineral mixture, each 1 kg of mixture content: 62.5 m I.U. Vit A, 25 m I.U. Vit. D_3_, 25 g Vit. E, 1.75 g Vit. K, 0.5 g Vit. B_1_, 2.75 g Vit. B_2_, 1.25 g Vit. B_6_, 10 mg. Vit. B_12_, 20 g niacin, 500 mg folic acid, 50 mg biotin, 37 g zinc, 22 g iron, 31 g manganese, 2.5 g copper, 50 mg cobalt, 113 mg selenium, 650 mg iodine.

⁣^b^Nitrogen free extract = 100 − (% crude protein + ether extract + crude fiber+ ash).

**Table 2:** Means of water quality variables of red hybrid tilapia fed on 0%, 1%, and 2% lemon balm leaves (LBL) under alternate-day feeding (ADF) and daily feeding (DF) for 60 days.

Parameters	Temperature (°C)	pH	DO (mg/L)	TAN (mg/L)
ADF + LBL0	22.35	7.92	4.56	0.33
ADF + LBL1	25.00	7.83	4.52	0.30
ADF + LBL2	24.80	7.84	5.41	0.33
DF + LBL0	25.00	7.80	4.60	0.27
DF + LBL1	24.77	7.85	6.80	0.28
DF + LBL2	24.9	7.68	4.70	0.23
PSE	0.14	0.03	0.29	0.01
*p*-Value	0.92	0.09	0.13	0.08

*Note:* ADF + LBL0, ADF + LBL1, and ADF + LBL2: fish fed a basal diet supplemented with 0%, 1%, and 2% LBL, respectively, under the ADF strategy. DF + LBL0, DF + LBL1, and DF + LBL2: fish were fed a basal diet supplemented with 0%, 1%, and 2% LBL, respectively, under the DF strategy. Two-way ANOVA (*n* = 3).

Abbreviations: DO, dissolved oxygen; PSE, pooled standard error; TAN, total ammonia nitrogen.

**Table 3:** Growth, feed utilization, and survival rate of red hybrid tilapia fed on 0%, 1%, and 2% lemon balm leaves (LBL) under alternate-day feeding (ADF) and daily feeding (DF) for 60 days.

Effect of the treatment	IBW (g)	FBW (g)	TWG (g/fish)	ADG (g/fish/day)	SGR (%/day)	FCR	SR (%)
Effect of feeding strategies							
ADF	18.63	28.48	9.85	0.16	0.69	1.68^b^	100
DF	16.98	27.20	10.22	0.17	0.76	2.36^a^	100
Effect of dietary LBL							
LBL0	17.18	26.23^b^	9.05^b^	0.15^b^	0.70^b^	2.24	100
LBL1	18.95	33.16^a^	14.20^a^	0.23^a^	0.93^a^	1.57	100
LBL2	17.29	24.15^b^	6.86^b^	0.11^b^	0.55^b^	2.26	100
Interaction effect (feeding strategies x dietary LBL)							
ADF + LBL0	17.7	25.50^b^	7.80^cd^	0.13^cd^	0.61^bc^	1.93^abc^	100
ADF +L BL1	19.91	33.30^a^	13.39^ab^	0.22^ab^	0.86^a^	1.31^c^	100
ADF + LBL2	18.28	26.66^b^	8.38^cd^	0.14^cd^	0.63^bc^	1.80^bc^	100
DF + LBL0	18.66	26.96^b^	10.30^bc^	0.17^bc^	0.79^ab^	2.53^ab^	100
DF + LBL1	18.00	33.02^a^	15.02^a^	0.25^a^	1.00^a^	1.82^bc^	100
DF + LBL2	16.30	21.64^b^	5.34^d^	0.09^d^	0.47^c^	2.72^a^	100
PSE	0.427	0.33	1.05	0.001	0.06	0.16	100

	***p*-Values**						

Two-way ANOVA							
Feeding strategies							
Dietary LBL	0.66	0.65	0.87	0.60	0.29	0.02	—
Interaction	0.13	0.01	0.01	<0.001	<0.001	0.04	—

*Note:* ADF + LBL0, ADF + LBL1, and ADF + LBL2: fish fed a basal diet supplemented with 0%, 1%, and 2% LBL, respectively, under the ADF strategy. DF + LBL0, DF + LBL1, and DF + LBL2: fish were fed a basal diet supplemented with 0%, 1%, and 2% LBL, respectively, under the DF strategy. The values carrying different superscript letters ^(a, b, c, d)^ in the same column are significantly different (*p* < 0.05; two-way ANOVA; *n* = 3).

Abbreviations: ADG, average daily gain; FBW, final body weight; FCR, feed conversion ratio; IBW, initial body weight; PSE, pooled standard error; SGR, specific growth rate; SR, survival rate; TWG, total weight gain.

**Table 4:** Body composition of red hybrid tilapia fed on 0%, 1%, and 2% lemon balm leaves (LBL) under alternate-day feeding (ADF) and daily feeding (DF) for 60 days.

Effect of the treatment	Moisture (%)	Crude lipid (%)	Crude protein (%)	Ash (%)
Effect of feeding strategies				
ADF	78.81	23.10	54.29	22.41
DF	77.93	22.86	55.76	21.47
Effect of dietary LBL				
LBL0	75.53	29.19^a^	52.03	18.79^c^
LBL1	80.61	23.05^b^	56.67	20.27^b^
LBL2	78.99	16.70^c^	56.38	26.76^a^
Interaction effect (feeding strategies x dietary LBL)				
ADF + LBL0	77.06	26.30^b^	54.56	19.31^bc^
ADF + LBL1	80.21	23.00^bc^	55.90	20.90^b^
ADF + LBL2	79.18	20.00^c^	52.41	27.03^a^
DF + LBL0	74.00	32.08^a^	49.51	18.28^c^
DF + LBL1	81.30	23.10^bc^	57.44	19.65^bc^
DF + LBL2	78.30	13.40^d^	60.35	26.50^a^
PSE	0.98	1.80	1.25	1.06

	***p*-Values**			

Two-way ANOVA				
Feeding strategies	0.67	0.95	0.53	0.68
Dietary LBL	0.09	0.03	0.26	<0.001
Interaction	0.41	<0.001	0.13	<0.001

*Note:* ADF + LBL0, ADF + LBL1, and ADF + LBL2: fish fed a basal diet supplemented with 0%, 1%, and 2% LBL, respectively, under the ADF strategy. DF + LBL0, DF + LBL1, and DF + LBL2: fish were fed a basal diet supplemented with 0%, 1%, and 2% LBL, respectively, under the DF strategy. The values carrying different superscript letters ^(a, b, c, d)^ in the same column are significantly different (*p* < 0.05; two-way ANOVA; *n* = 3).

Abbreviation: PSE, pooled standard error.

**Table 5:** Hematological variables of red hybrid tilapia fed on 0%, 1%, and 2% lemon balm leaves (LBL) under alternate-day feeding (ADF) and daily feeding (DF) for 60 days.

Effect of the treatment	RBCs (10^6^/mm^3^)	Hb (g/dL)	Hct (%)	WBCs (10^3^/mm^3^)
Effect of feeding strategies				
ADF	2.56	7.65	23.53	152.17
DF	2.70	7.90	24.50	325.67
Effect of dietary LBL				
LBL0	2.45	7.37^b^	22.50^b^	138.78^b^
LBL1	2.55	7.65^b^	23.25^b^	159.00^b^
LBL2	2.90	8.30^a^	26.25^a^	419.44^a^
Interaction effect (feeding strategies x dietary LBL)				
ADF + LBL0	2.40	7.35^b^	22.60^b^	137.55^b^
ADF + LBL1	2.50	7.50^b^	22.50^b^	161.00^b^
ADF + LBL2	2.80	8.10^a^	25.50^a^	158.88^b^
DF + LBL0	2.50	7.40^b^	22.50^b^	140.00^b^
DF + LBL1	2.60	7.80^ab^	24.00^ab^	157.00^b^
DF + LBL2	3.00	8.50^a^	27.00^a^	680.00^a^
PSE	0.08	0.14	0.56	59.66

	***p*-Values**			

Two-way ANOVA				
Feeding strategies	0.48	0.40	0.41	0.15
Dietary LBL	0.41	0.03	0.04	0.02
Interaction	0.45	0.05	0.01	<0.001

*Note:* ADF + LBL0, ADF + LBL1, and ADF + LBL2: fish fed a basal diet supplemented with 0%, 1%, and 2% LBL, respectively, under the ADF strategy. DF + LBL0, DF + LBL1, and DF + LBL2: fish were fed a basal diet supplemented with 0%, 1%, and 2% LBL, respectively, under the DF strategy. The values carrying different superscript letters ^(a, b)^ in the same column are significantly different (*p* < 0.05; two-way ANOVA; *n* = 3).

Abbreviations: Hb, hemoglobin; Hct, hematocrit; PSE, pooled standard error; RBCs, red blood cells; WBCs, white blood cells.

**Table 6:** Biochemical variables of red hybrid tilapia fed on 0%, 1%, and 2% lemon balm leaves (LBL) under alternate-day feeding (ADF) and daily feeding (DF) for 60 days.

Effect of the treatment	ALT (U/L)	AST (U/L)	Glucose (ng/L)	Cholesterol (mg/dL)	Alpha-antitrypsin (mg/dL)
Effect of feeding strategies					
ADF	7.66	8.00	47.83^b^	203.50	97.67
DF	6.50	7.50	61.00^a^	192.33	104.16
Effect of dietary LBL					
LBL0	7.50	9.25	55.25	197.00	94.00
LBL1	9.25	8.00	53.75	195.25	98.00
LBL2	4.50	6.00	54.25	201.50	111.50
Interaction effect (feeding strategies x dietary LBL)					
ADF + LBL0	9.00	8.50	45.50^b^	198.00	95.00
ADF + LBL1	10.00	9.00	47.50^b^	202.50	98.50
ADF + LBL2	4.00	6.50	50.50^b^	210.00	100.00
DF + LBL0	6.00	10.00	65.00^a^	196.00	93.00
DF + LBL1	8.50	7.00	60.00^a^	188.00	97.50
DF + LBL2	5.00	5.50	58.00^ab^	193.00	122.00
PSE	0.77	0.66	2.19	2.48	3.01

	***p*-Values**				

Two-way ANOVA					
Feeding strategies	0.48	0.72	<0.001	0.01	0.33
Dietary LBL	0.09	0.13	0.98	0.79	0.39
Interaction	0.08	0.42	<0.001	0.10	0.12

*Note:* ADF + LBL0, ADF + LBL1, and ADF + LBL2: fish fed a basal diet supplemented with 0%, 1%, and 2% LBL, respectively, under the ADF strategy. DF + LBL0, DF + LBL1, and DF + LBL2: fish were fed a basal diet supplemented with 0%, 1%, and 2% LBL, respectively, under the DF strategy. The values carrying different superscript letters ^(a, b)^ in the same column are significantly different (*p* < 0.05; two-way ANOVA; *n* = 3).

Abbreviations: ALT, alanine aminotransferase; AST, aspartate aminotransferase; PSE, pooled standard error.

**Table 7:** Digestive enzyme activity of red hybrid tilapia fed on 0%, 1%, and 2% lemon balm leaves (LBL) under alternate-day feeding (ADF) and daily feeding (DF) for 60 days.

Effect of the treatment	Lipase (U/g protein)	Amylase (U/g protein)
Effect of feeding strategies		
ADF	21.00	24.66^b^
DF	24.30	47.66^a^
Effect of dietary LBL		
LBL0	21.00	30.00
LBL1	22.50	34.50
LBL2	24.50	44.00
Interaction effect (feeding strategies x dietary LBL)		
ADF + LBL0	20.00^b^	22.00^c^
ADF + LBL1	21.00^b^	25.00^b^
ADF + LBL2	22.50^b^	27.00^b^
DF + LBL0	22.00^b^	38.00^ab^
DF + LBL1	24.00^a^	44.00^ab^
DF + LBL2	27.00^a^	61.00^a^
PSE	1.57	4.09

	***p*-Values**	

Two-way ANOVA		
Feeding strategies	0.10	<0.001
Dietary LBL	0.18	0.73
Interaction	<0.001	<0.001

*Note:* ADF + LBL0, ADF + LBL1, and ADF + LBL2: fish fed a basal diet supplemented with 0%, 1%, and 2% LBL, respectively, under the ADF strategy. DF + LBL0, DF + LBL1, and DF + LBL2: fish were fed a basal diet supplemented with 0%, 1%, and 2% LBL, respectively, under the DF strategy. The values carrying different superscript letters ^(a, b, c)^ in the same column are significantly different (*p* < 0.05; two-way ANOVA; *n* = 3).

Abbreviation: PSE, pooled standard error.

## Data Availability

Data are available upon a reasonable request from the corresponding author.
